# Pan-cancer assessment of antineoplastic therapy-induced interstitial lung disease in patients receiving subsequent therapy immediately following immune checkpoint blockade therapy

**DOI:** 10.1186/s12931-024-02683-8

**Published:** 2024-01-10

**Authors:** Yoshihiro Kitahara, Yusuke Inoue, Hideki Yasui, Masato Karayama, Yuzo Suzuki, Hironao Hozumi, Kazuki Furuhashi, Noriyuki Enomoto, Tomoyuki Fujisawa, Kazuhito Funai, Tetsuya Honda, Kiyoshi Misawa, Hideaki Miyake, Hiroya Takeuchi, Naoki Inui, Takafumi Suda

**Affiliations:** 1 Second Division, Department of Internal Medicine, Hamamatsu University School of Medicine, 1-20-1 Handayama, Higashi-ku, Hamamatsu, 431-3192 Japan; 2 Department of Chemotherapy, Hamamatsu University School of Medicine, 1-20-1 Handayama, Higashi-ku, Hamamatsu, 431-3192 Japan; 3 First Department of Surgery, Hamamatsu University School of Medicine, 1-20-1 Handayama, Higashi-ku, Hamamatsu, 431-3192 Japan; 4 Department of Dermatology, Hamamatsu University School of Medicine, 1-20-1 Handayama, Higashi-ku, Hamamatsu, 431-3192 Japan; 5 Department of Otorhinolaryngology, Hamamatsu University School of Medicine, 1-20-1 Handayama, Higashi-ku, Hamamatsu, 431-3192 Japan; 6 Department of Urology, Hamamatsu University School of Medicine, 1-20-1 Handayama, Higashi-ku, Hamamatsu, 431-3192 Japan; 7 Department of Surgery, Hamamatsu University School of Medicine, 1-20-1 Handayama, Higashi-ku, Hamamatsu, 431-3192 Japan; 8 Department of Clinical Pharmacology and Therapeutics, Hamamatsu University School of Medicine, 1-20-1 Handayama, Higashi-ku, Hamamatsu, 431-3192 Japan

**Keywords:** Pneumonitis, Interstitial lung disease, Docetaxel, Immune checkpoint inhibitor, Drug-induced pneumonitis

## Abstract

**Background:**

Drug-induced interstitial lung disease (DIILD) is a serious adverse event potentially induced by any antineoplastic agent. Whether cancer patients are predisposed to a higher risk of DIILD after receiving immune checkpoint inhibitors (ICIs) is unknown.

**Methods:**

This study retrospectively assessed the cumulative incidence of DIILD in consecutive cancer patients who received post-ICI antineoplastic treatment within 6 months from the final dose of ICIs. There was also a separate control cohort of 55 ICI-naïve patients with non-small cell lung cancer (NSCLC) who received docetaxel.

**Results:**

Of 552 patients who received ICIs, 186 met the inclusion criteria. The cohort predominantly comprised patients with cancer of the lung, kidney/urinary tract, or gastrointestinal tract. The cumulative incidence of DIILD in the entire cohort at 3 and 6 months was 4.9% (95% confidence interval [CI] 2.4%–8.7%) and 7.2% (95% CI 4.0%–11.5%), respectively. There were significant differences according to cancer type (Gray’s test, *P* = .04), with the highest cumulative incidence of DIILD in patients with lung cancer being 9.8% (95% CI 4.3%–18.0%) at 3 months and 14.2% (95% CI 7.3%–23.3%) at 6 months. DIILD was caused by docetaxel in six of these 11 lung cancer patients (54.5%). After matching, the cumulative incidence of docetaxel-induced ILD in patients with NSCLC in the post-ICI setting was higher than that in the ICI-naïve setting: 13.0% (95% CI 3.3%–29.7%) vs 4.3% (95% CI 0.3%–18.2%) at 3 months; and 21.7% (95% CI 7.9%–39.9%) vs 4.3% (95% CI 0.3%–18.2%) at 6 months. However, these were not significant differences (hazard ratio, 5.37; 95% CI 0.64–45.33; Fine–Gray* P* = .12).

**Conclusions:**

Patients with lung cancer were at high risk of developing DIILD in subsequent regimens after ICI treatment. Whether NSCLC patients are predisposed to additional risk of docetaxel-induced ILD by prior ICIs warrants further study.

**Supplementary Information:**

The online version contains supplementary material available at 10.1186/s12931-024-02683-8.

## Background

Since the first approval of ipilimumab in 2011, immunotherapy with immune checkpoint inhibitors (ICIs) targeting programmed death-1 (PD-1), its ligand (PD-L1), and cytotoxic T-lymphocyte-associated antigen 4 (CTLA-4), either as monotherapy, immunochemotherapy, or dual immuno-oncology (IO) combination therapy, has transformed the treatment landscape of many cancer types by offering durable responses in a subset of patients. With the growing interest in ICIs, the number of indications is increasing substantially, and ICIs are approved across nearly 20 different cancers. As of 2018, more than 40% of cancer patients in the United States were considered eligible for ICIs [1].

With the expansion of ICI use, management of immune-related adverse events (irAEs) has been an intense focus. Among a wide range of irAEs, ICI-induced interstitial lung disease (ILD) is a serious toxicity leading to interruption or cessation of treatment and worse survival outcomes in patients receiving immunotherapy [2]. We previously reported that the incidence of ICI-induced ILD in the real-world was higher than that reported in clinical trials in patients with non-small cell lung cancer (NSCLC) [3, 4], and many other studies have been conducted to reveal the features, risk factors, and prognosis of ICI-induced ILD [3, 5–8]. Importantly, increased activity of immune responses induced by ICIs can increase the risk of ILD in patients with NSCLC carrying epidermal growth factor receptor (*EGFR*) mutations, not only when EGFR tyrosine kinase inhibitors, particularly osimertinib, were administered in combination with PD-1/PD-L1 inhibitors [9–11], but also when they were administered sequentially [9, 12]. The similar influence of prior immunotherapy on the frequency of immune-mediated reactions to subsequent kinase inhibitors has been observed in several oncogene-driven NSCLCs and melanoma [13–17]. However, although drug-induced ILD (DIILD) could be caused by any type of antineoplastic agent [18], it remains unknown whether the incidence and severity of DIILD are affected by immediately prior ICI therapy. This clinical question is becoming more crucial because upfront IO therapies using ICIs have become the standard of care for multiple types of cancers, and cancer patients are therefore more likely than before to be exposed to subsequent antineoplastic agents after receiving ICIs. These subsequent antineoplastic agents include a wide range of therapeutics, such as chemotherapy, molecular targeted therapy, monoclonal antibodies, and antibody–drug conjugates, some of which (e.g., trastuzumab deruxtecan [19]) are known to be associated with a substantial risk of ILD.

In this study, we conducted a pan-cancer analysis to investigate the cumulative incidence, characteristics, and risk factors of DIILD in cancer patients in the setting of subsequent antineoplastic treatment following prior regimens containing ICIs.

## Methods

### Study design and patients

In this retrospective, single-center, pan-cancer study, we collected the clinical data of consecutive patients with pathologically diagnosed advanced or recurrent cancer (lung, pleura, kidney, urinary tract, skin, head and neck, parathyroid, gastrointestinal tract, and unknown primary organ) who had received ICI therapy between September 2014 and July 2022 at Hamamatsu University Hospital, Japan. Data were analyzed from December 2022 to February 2023. We selected patients aged ≥ 20 years who had received subsequent systemic antineoplastic treatment that immediately followed regimens containing ICIs, and the cumulative incidence and features of DIILD were assessed in this treatment setting. Patients for whom subsequent systemic antineoplastic treatment was initiated after more than 6 months from the final dose of ICIs were excluded [20] because longer intervals, especially more than 6 months, from the last dose of ICIs would make the potential influence of prior ICIs on adverse events in subsequent regimens elusive [21]. If patients received multiple lines of ICI treatment in a non-consecutive manner, DIILD in a subsequent regimen following former ICIs was assessed. When patients had received multiple ICIs in a consecutive manner, DIILD in a subsequent regimen following the latter ICIs was assessed.

After identifying lung cancer patients, particularly those who received docetaxel, as possessing a higher risk of DIILD, we collected clinical data of consecutive ICI-naïve patients with NSCLC treated with docetaxel-containing regimens between May 2012 and July 2022 at Hamamatsu University Hospital as a control. The cumulative incidence of DIILD caused by docetaxel in those patients was calculated and compared with that in the post-ICI setting.

### Data collection

Clinical data at the time of initiation of post-ICI antineoplastic treatment, including age, sex, smoking status, comorbidities, Eastern Cooperative Oncology Group performance status (ECOG-PS), treatment line, regimens, adverse events, and laboratory data, were obtained from the review of electronic medical records. Original pathological information of tumors and cancer stages were also reviewed.

### Diagnosis of DIILD

DIILD during treatment with post-ICI systemic antineoplastic agents was defined as newly appearing interstitial findings in the lungs confirmed on high-resolution computed tomography (HRCT) during treatment or within 30 days of the last treatment administration. Bilateral reticular or ground-glass opacities and consolidation affecting at least 10% of lung volume on HRCT was considered compatible with ILD. The threshold of 10% was provided to exclude patients with minimal abnormality and minimal clinical impact. ILD was diagnosed by physicians and board-certified radiologists along with board-certified pulmonologists (Y.K. and Y.I.). ILDs that could be explained by other causes such as non-antineoplastic drugs, infection, pulmonary edema, radiation pneumonitis, and lymphangitic carcinomatosis of the lungs were excluded. ILD patterns on HRCT images with 1.25-mm-thick sections, including nonspecific interstitial pneumonia (NSIP), organizing pneumonia (OP), hypersensitivity pneumonia (HP), diffuse alveolar damage (DAD), and simple pulmonary eosinophilia (PEo) patterns, were evaluated according to the Fleischner Society's position paper [22]. The severity of DIILD was evaluated according to the Common Terminology Criteria for Adverse Events version 5.0.

### Statistical analysis

Fisher’s exact test and Mann–Whitney U test were used to compare characteristics between two groups, and the distributions of clinical factors were summarized as frequency (%) or median (range). Median follow-up time was estimated by the reverse Kaplan–Meier method. The Gray’s test was used to estimate and compare the cumulative incidence of DIILD, and a hazard ratio (HR) with 95% confidence interval (CI) was calculated using Fine–Gray’s competing-risks regression. Competing risks were defined as death due to any cause and discontinuation of post-ICI antineoplastic treatment due to any reason other than DIILD. The date of treatment discontinuation was defined as 30 days after the last dose of post-ICI antineoplastic treatment, or the date of initiation of new systemic therapy after the last dose of post-ICI treatment, whichever occurred earlier [4].

To minimize bias in an attempt to verify results in the comparison of cumulative incidence of docetaxel-induced ILD in NSCLC patients between post-ICI and ICI-naïve settings, propensity score matching was performed to create two comparable groups of patients. The propensity score for each patient was estimated with a logistic regression model that included age (<75 years versus ≥75 years), sex, ECOG-PS (≤1 versus ≥2), and concomitant use of vascular endothelial growth factor (VEGF)/VEGF-receptor (R) inhibitors. The nearest-neighbor matching method with a 1:1 matching ratio was used without replacement, with a caliper of 0.2.

All analyses were performed using EZR statistical software [23] version 1.55 (Saitama Medical Center, Jichi Medical University, Saitama, Japan). Statistical tests were two-sided, and a significance level was set at a *P*-value of < .05 for all analyses.

## Results

### Characteristics of the study cohort

**Fig. 1:**
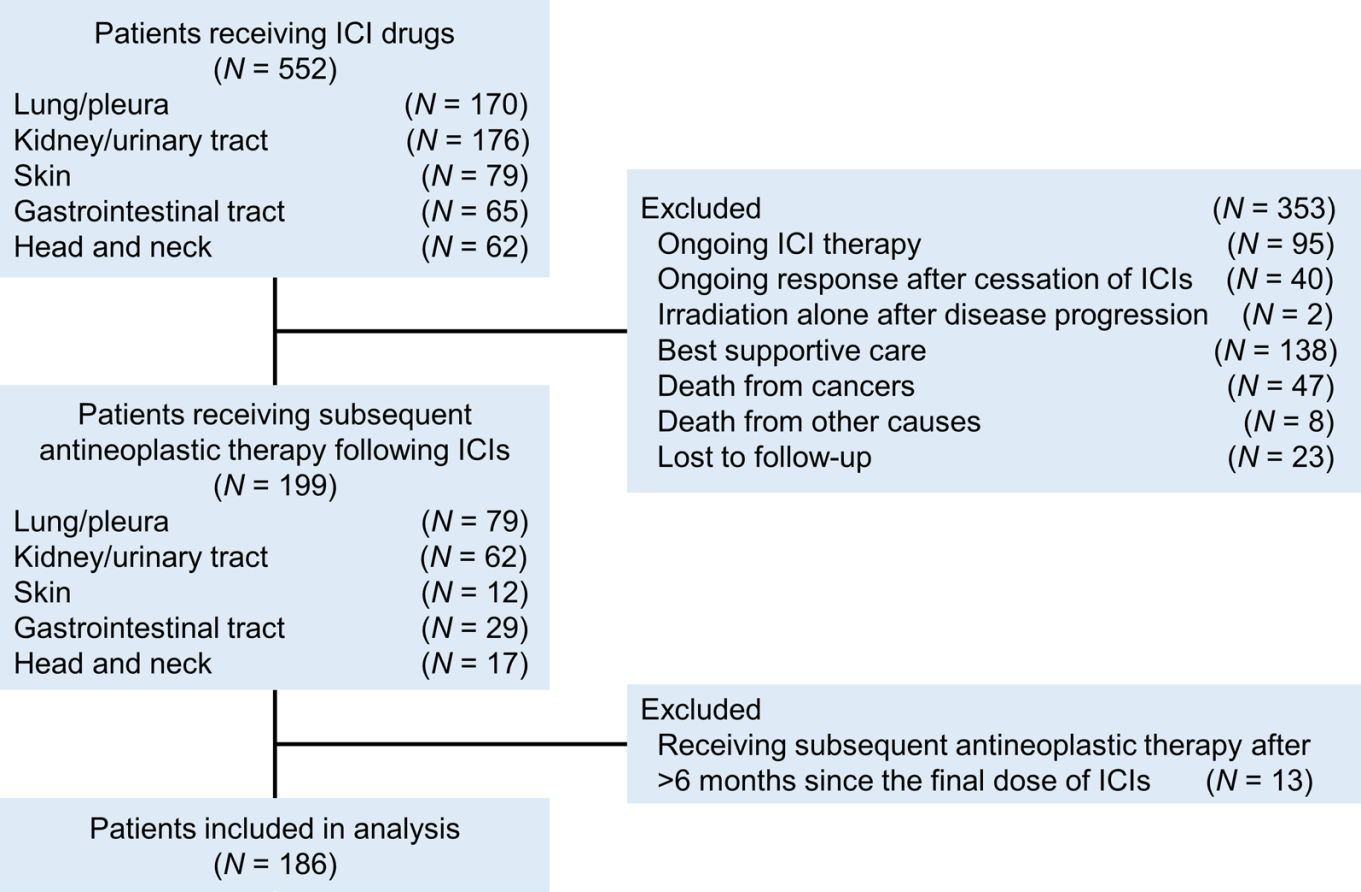
Study flow diagram. ICI, immune checkpoint inhibitor; irAEs, immune-related adverse events

ICIs were used for 552 cancer patients during the study period, among whom 199 patients were treated with subsequent systemic therapy that immediately followed ICI-containing regimens. Thirteen patients who had received subsequent therapy >6 months after the final dose of ICIs were excluded, resulting in 186 assessable patients (Fig. 1). No patients received concurrent thoracic radiotherapy with post-ICI treatment. The characteristics of the cohort at the time of initiation of post-ICI treatment (baseline) are shown in Table 1. The median age was 68 (range, 32–83) years. Male patients (*N* = 143, 76.9%), patients with ECOG-PS of 0 or 1 (*N* = 134, 72.0%), and patients with a smoking history (*N* = 130, 70.0%) were predominant. The cohort mainly comprised patients with lung cancer (*N* = 73, 39.2%; NSCLC, *N* = 69, 37.1%; SCLC, *N* = 4, 2.2%); kidney/urinary tract cancer (*N* = 58, 31.2%); and gastrointestinal cancer (*N* = 28, 15.1%). Preexisting ILD at the time of initiation of prior ICI therapy was identified in 25 patients (13.4%). Thoracic radiotherapy was performed before baseline in 34 patients (18.3%). Prior ICI therapies were PD-1/PD-L1 inhibitor monotherapy (*N* = 126, 67.7%), immunochemotherapy (*N* = 39, 21.0%), combination with molecular targeted agents (*N* = 14, 7.5%), and combined PD-1/PD-L1 and CTLA-4 blockade therapy with or without chemotherapy (*N* = 7, 3.8%). Prior ICI-containing therapies were discontinued because of disease progression (*N* = 145, 78.0%), adverse events (*N* = 33, 17.7%), or deterioration of general condition (*N* = 3, 1.6%). Prior ICI-induced ILD developed in 18 patients (9.7%), resulting in ICI discontinuation in 16 of these patients (88.9%). The median interval between the last ICI administration and the initiation of post-ICI treatment was 1.6 (range, 0.5–5.9) months.

**Table 1:** Patient characteristics

	Patients, No. (%)
	All patients (*N* = 186)
Age, median (range), y	68 (32–83)
Sex	
Male	143 (76.9)
Female	43 (23.1)
ECOG-PS	
0 or 1	134 (72.0)
≥2	52 (28.0)
Smoking status	
Never	36 (19.4)
Current or former	130 (70.0)
Unknown	20 (10.8)
Stage	
III	29 (15.6)
IV	116 (62.4)
Recurrence	41 (22.0)
Primary organ	
Lung/pleura	74 (39.8)
Kidney/urinary tract	58 (31.2)
Skin	10 (5.4)
Gastrointestinal tract	28 (15.1)
Head and neck	16 (8.6)
History of prior thoracic radiotherapy	34 (18.3)
Prior PD-1/PD-L1 inhibitors	
Nivolumab	89 (47.8)
Pembrolizumab	64 (34.4)
Atezolizumab	22 (11.8)
Durvalumab	6 (3.2)
Avelumab	5 (2.7)
Prior ICI treatment	
Monotherapy	126 (67.7)
Immunochemotherapy	39 (21.0)
Combination with CTLA-4 blockade therapy with or without chemotherapy	7 (3.8)
Combination with molecular targeted agents	14 (7.5)
irAEs in prior ICI regimens	
ICI-induced ILD	18 (9.7)
Others	62 (33.3)
Duration of ICI therapy, median (range), m	4.0 (0.5–39.9)
Post-ICI antineoplastic therapy	
Cytotoxic chemotherapy alone	106 (57.0)
Molecular targeted therapy with or without cytotoxic agents^a^	80 (43.0)
Treatment line	
2nd	69 (37.1)
3rd	61 (32.8)
≥4th	56 (30.1)
Duration of post-ICI antineoplastic therapy, median (range), m	3.0 (0.5–39.9)

^a^Twenty-three patients were treated with molecular targeted therapy combined with cytotoxic agents including antibody–drug conjugates

CTLA-4: cytotoxic T-lymphocyte-associated antigen 4; ECOG: Eastern Cooperative Oncology Group; ICIs: immune checkpoint inhibitors; ILD: interstitial lung disease; irAEs: immune-related adverse events; PD-1: programmed death-1; PD-L1: programmed death-ligand 1; PS: performance status

### Characteristics of DIILD

DIILD during post-ICI antineoplastic therapy was identified in 14 patients (7.5%), including 11 patients with lung cancer (10 with NSCLC and 1 with SCLC) and 1 with bladder, esophageal, or buccal mucosa cancer each. There was no significant difference in patient characteristics between patients with or without DIILD, except for the presence of pleural effusion, which was more commonly observed in patients with DIILD (71.4% vs 36.0%, *P* = .02; Additional file 5: Table S1). Although preexisting ILD could be considered a risk factor for the development DIILD, there was no significant difference in the prevalence of ILD between patients with or without DIILD (21.4% vs 12.8%, *P* = .41; Additional file 5: Table S1). Representative chest CT images of the three patients with preexisting ILD who developed DIILD are shown in Additional file 1: Figure S1 both before initiation of post-ICI antineoplastic therapy and at the time of DIILD diagnosis. These patients were diagnosed with DIILD instead of exacerbation of preexisting ILD because of changes in radiological patterns of newly developed abnormalities, and the time of appearance of new interstitial findings in the lungs. The detailed information of patients with DIILD is shown in Additional file 6: Table S2, and antineoplastic agents that were considered to cause DIILD are listed in Additional file 7: Table S3. The median time from treatment initiation to the onset of DIILD was 63 (range, 6–201) days. Docetaxel was the most common agent to be considered to cause ILD (7 of 14, 50%). Of the 14 patients with DIILD, radiological ILD patterns were classified as OP in 5 patients (35.7%), HP in 4 patients (28.6%), DAD in 4 patients (28.6%), and PEo in 1 patient (7.1%). The severity of DIILD was grade 1 or 2 in six patients, grade 3 in five patients, and grade 5 in three patients (due to osimertinib [*N* = 2] and docetaxel plus ramucirumab [*N* = 1]). Among 18 patients who had developed prior ICI-induced ILD, three (16.7%) again developed DIILD at the post-ICI setting and one of them died from DIILD caused by docetaxel plus ramucirumab (Additional file 6: Table S2). Among the surviving patients, seven responded to corticosteroids, while four recovered without corticosteroids after discontinuation of the treatment regimen.

### Cumulative incidence of DIILD

**Fig. 2:**
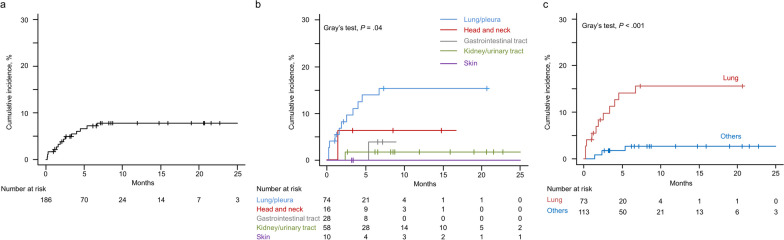
Cumulative incidence of drug-induced interstitial lung disease (DIILD) in cancer patients receiving subsequent antineoplastic therapy following prior immune checkpoint inhibitor-containing regimens. **a** Cumulative incidence of DIILD in the entire pan-cancer cohort. **b** Cumulative incidence of DIILD according to cancer type. **c** Cumulative incidence of DIILD in patients with lung cancer or other cancer types

The median follow-up time since the initiation of post-ICI treatment was 13.9 (95% CI 10.0–17.8) months. The cumulative incidence of DIILD caused by post-ICI antineoplastic agents at 1, 2, 3, and 6 months after treatment initiation was 1.6% (95% CI 0.4–4.3%), 3.8% (95% CI 1.7–7.3%), 4.9% (95% CI 2.4–8.7%), and 7.2% (95% CI 4.0–11.5%), respectively (Fig. 2a). There were significant differences according to cancer type (*P* = .04), with the highest cumulative incidence of DIILD being observed in patients with lung cancer (Fig. 2b). When divided into the two groups (lung and other cancers), the cumulative incidence of DIILD was significantly higher in patients with lung cancer (*P* < .001), with that at 1, 2, 3, and 6 months being 4.1% vs 0.9%, 8.2% vs 0.9%, 9.7% vs 1.8%, and 14.0% vs 2.7%, respectively (Fig. 2c). There was no significant difference in cumulative incidence of DIILD between treatment types (cytotoxic chemotherapy alone vs molecular targeted therapy; Additional file 2: Fig. S2).

Fine–Gray analyses for predicting DIILD caused by post-ICI treatment are shown in Table 2. In univariable analyses, a history of ICI-induced ILD (HR, 3.99; 95% CI 1.29–12.39; *P* = .02), lung cancer (HR, 6.19; 95% CI 1.75–21.91; *P* = .005), and the presence of pleural fluid (HR, 4.15; 95% CI 1.31–13.16; *P* = .02) were significantly associated with DIILD. When adjusted for ICI-induced ILD history, lung cancer alone remained significantly associated with DIILD (HR, 5.33; 95% CI 1.52–18.75; *P* = .009). Similarly, when adjusted for the presence of pleural fluid, lung cancer alone also remained significantly associated with DIILD (HR, 4.72; 95% CI 1.38–16.11; *P* = .01).

**Table 2:** Univariable and two-variable Fine-Gray analyses for prediction of drug-induced interstitial lung disease

Variable	Univariable			Two-variable model								
				Model 1			Model 2			Model 3		
	HR	95% CI	*P* value	HR	95% CI	*P* value	HR	95% CI	*P* value	HR	95% CI	*P* value
Age (≥75 vs <75 years)	0.25	0.03–1.88	.18									
Sex (male vs female)	1.89	0.43–8.39	.40									
Smoking history (ever vs never)	1.01	0.99–1.03	.60									
ECOG-PS (≥2 vs <2)	0.97	0.30–3.12	.96									
History of ILD (yes vs no)	1.58	0.21–11.67	.66									
History of ICI-induced ILD (yes vs no)	3.99	1.29–12.39	.02	2.61	0.87–7.88	.08						
History of prior thoracic radiotherapy (yes vs no)	1.22	0.35–4.26	.76							0.78	0.23–2.67	.69
History of irAEs (yes vs no)	1.02	0.36–2.92	.97									
PD-L1 expression (≥50% vs <50%)	2.03	0.50–8.33	.32									
Lung cancer (yes vs no)	6.19	1.75–21.91	.005	5.33	1.52–18.75	.009	4.72	1.38–16.11	.01	6.45	1.83–22.69	.004
Interval between ICI therapy and post-ICI antineoplastic therapy (≥3 months vs <3 months)	2.57	0.81–8.14	.11									
Presence of pleural fluid (yes vs no)	4.15	1.31–13.16	.02				2.81	0.91–8.71	.07			
Presence of pulmonary emphysema (yes vs no)	1.93	0.67–5.55	.22									
Response to prior ICI therapy (yes vs no)	2.8	0.92–8.50	.06									
Treatment line (≥4th vs ≤3rd)	1.03	0.32–3.32	.97									

CI: confidence interval; ECOG: Eastern Cooperative Oncology Group; HR: hazard ratio; ICI: immune checkpoint inhibitor; ILD: interstitial lung disease; irAEs: immune-related adverse events; PD-L1: programmed death ligand 1; PS: performance status

Because lung cancer was identified as an independent factor associated with DIILD, we next compared the patients’ characteristics with lung cancer and other cancers (Additional file 8: Table S4). Some comorbidities including hypertension (45.2% vs 29.2%; *P* = .03), chronic obstructive pulmonary disease (19.2% vs 5.3%; *P* = .006), and pleural fluid (56.2% vs 27.4%; *P* < .001) were significantly more common in the lung cancer group, whereas preexisting ILD was significantly less common in this group (6.8% vs 17.7%; *P* = .046). The proportion of patients with a history of prior thoracic radiotherapy was significantly higher in lung cancer patients (28.8% vs 11.5%; *P* = .004). Again, however, lung cancer alone remained significantly associated with the development of DIILD (HR, 6.45; 95% CI 1.83–22.69; Fine–Gray* P* = .004) when adjusted for the history of prior thoracic radiotherapy (Table 2).

### Comparison of docetaxel-induced ILD in NSCLC patients between the post-ICI and ICI-naïve settings

**Fig. 3:**
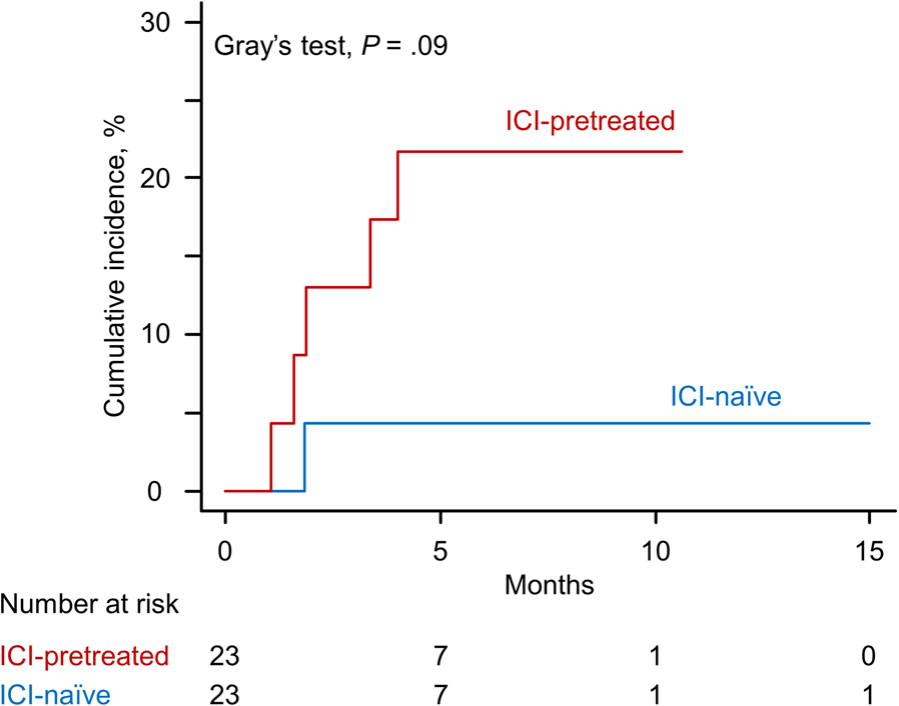
Cumulative incidence function for the risk of developing docetaxel-induced interstitial lung disease in propensity score-matched patients with non-small cell lung cancer at the post-immune checkpoint inhibitor (ICI) or ICI-naïve setting

In lung cancer patients, docetaxel-containing regimens were most frequently selected as post-ICI treatments (28 of 73 patients [38.4%]). Notably, six of 11 cases (54.5%) of DIILD in patients with lung cancer were caused by docetaxel, all of which had NSCLC histology. There were four NSCLC patients who had been treated with durvalumab after chemoradiotherapy for stage III disease, and ILD was developed in one of these patients by docetaxel (Additional file 6: Table S2). The cumulative incidence of docetaxel-induced ILD was numerically higher compared with that of ILD caused by other cytotoxic agents, but there was no significant difference (*P* = .13; Additional file 3: Fig. S3a). Similarly, there was no significant difference in cumulative incidence of DIILD between patients who received taxane-based or non-taxane-based regimens (*P* = .09; Additional file 3: Fig. S3b). To explore whether prior ICI treatment affects the incidence of docetaxel-induced ILD in patients with NSCLC, we next collected the clinical data of consecutive ICI-naïve NSCLC patients who were treated with docetaxel-containing regimens as a control cohort (*N* = 55). This cohort included four patients with stage III disease who had been treated with platinum-based chemotherapy concurrently with definitive radiation therapy before receiving docetaxel. The characteristics of patients receiving docetaxel at either the post-ICI or ICI-naïve setting are shown in Table 3. In the ICI-naive cohort, docetaxel-induced ILD was identified in four patients (7.3%). The severities of ILD were grade 1, 2, 3, or 5 in one patient each. Two patients responded to corticosteroids and one recovered after discontinuation of docetaxel without specific treatment. One patient died after high-dose corticosteroid treatment. While not statistically significant, the cumulative incidence of docetaxel-induced ILD was numerically higher in ICI-pretreated patients than in ICI-naïve patients (HR, 2.99; 95% CI 0.86–10.36; Fine–Gray* P* = .09), with that being 14.3% vs 5.6% at 3 months and 21.4% vs 7.6% at 6 months (Additional file 4: Fig. S4). After matching (23 patients per group; Table 3), the difference in the cumulative incidence of docetaxel-induced ILD between both groups appeared clinically meaningful, but remained not statistically significant (HR, 5.37; 95% CI 0.64–45.33; Fine–Gray* P* = .12), with that being 13.0% vs 4.3% at 3 months and 21.7% vs 4.3% at 6 months (Fig. 3).

**Table 3:** Characteristics of patients with non-small cell lung cancer treated with docetaxel with or without prior ICI treatment

	Patients, No. (%)					
	Full study population			Propensity score-matched cohort		
	Post-ICI	ICI-naïve	*P*-value	Post-ICI	ICI-naïve	*P*-value
	(*N* = 28)	(*N* = 55)		(*N* = 23)	(*N* = 23)	
Age, median (range), y	67 (43–79)	65 (33–83)	.18	66 (43–79)	65 (44–83)	.82
Sex						
Male	22 (78.6)	40 (72.7)	.61	17 (73.9)	17 (73.9)	>.99
Female	6 (21.4)	15 (27.3)		6 (26.1)	6 (26.1)	
ECOG-PS						
0 or 1	23 (82.1)	46 (72.1)	>.99	19 (82.6)	18 (78.3)	>.99
≥2	5 (17.8)	9 (27.9)		4 (17.4)	5 (21.7)	
Smoking status						
Never	4 (14.3)	13 (23.6)	.39	3 (13.0)	7 (30.4)	.16
Current or former	24 (85.7)	40 (72.7)		20 (87.0)	15 (65.2)	
Unknown	0 (0)	2 (3.6)			1 (4.3)	
Histological subtype						
Adenocarcinoma	21 (75.0)	37 (67.3)	.82	16 (69.6)	17 (73.9)	>.99
Squamous cell carcinoma	4 (14.3)	11 (20.0)		4 (17.4)	3 (13.0)	
Other^a^	3 (10.7)	7 (12.7)		3 (13.0)	3 (13.0)	
Comorbidity						
Hypertension	12 (42.9)	17 (30.9)	.33	9 (39.1)	9 (39.1)	>.99
Diabetes mellitus	7 (25.0)	8 (14.5)	.36	5 (21.7)	6 (26.1)	>.99
COPD	3 (10.7)	6 (10.9)	>.99	3 (13.0)	3 (13.0)	>.99
Pulmonary emphysema	13 (46.4)	23 (41.8)	.81	11 (47.8)	9 (39.1)	.76
Pleural fluid	16 (57.2)	19 (34.5)	.06	14 (60.9)	10 (43.5)	.37
ILD	2 (7.1)	5 (9.1)	>.99	2 (8.7)	3 (13.0)	>.99
Radiation pneumonitis	3 (10.7)	5 (9.1)	>.99	3 (13.0)	1 (4.3)	.61
Cardiovascular disease	2 (7.1)	10 (18.2)	.32	2 (8.7)	3 (13.0)	>.99
Renal disorder	0 (0)	2 (3.6)	.54	0 (0)	2 (8.7)	.48
Stage						
III	5 (17.2)	13 (23.6)	.58	5 (21.7)	4 (17.4)	.43
IV	20 (69.0)	38 (69.1)		17 (73.9)	15 (65.2)	
Recurrence	3 (10.7)	4 (7.3)		1 (4.3)	4 (17.4)	
History of prior thoracic radiotherapy	6 (21.4)	10 (18.2)	.77	6 (26.1)	2 (8.7)	.24
irAEs in prior ICI therapy						
ICI-induced ILD	2 (7.1)	NA		1 (4.3)	NA	
Others	12 (42.9)			10 (43.5)		
Docetaxel treatment						
Monotherapy	10 (35.7)	29 (52.7)	.004	10 (43.5)	10 (43.5)	>.99
With VEGF/VEGF-R inhibitors^b^	17 (60.7)	14 (25.5)		12 (52.2)	12 (52.2)	
With cisplatin	1 (3.6)	12 (21.8)		1 (4.3)	1 (4.3)	
Treatment line						
≤3rd	25 (89.3)	48 (87.3)	>.99	21 (91.3)	18 (78.3)	.41
≥4th	3 (10.7)	7 (12.7)		2 (8.7)	5 (21.7)	
Duration of docetaxel therapy, median (range), m	2.0 (0.5–10.1)	2.6 (0.5–22.1)	.95	2.1 (0.5–10.1)	1.6 (0.5–22.1)	.86
Total cycle of docetaxel, median (range)	3 (1–14)	4 (1–31)	.91	3 (1–13)	3 (1–31)	.93

^a^Other histology includes NSCLC not otherwise specified, large cell neuroendocrine carcinoma, and adenosquamous carcinoma

^b^VEGF/VEGF-R inhibitors include ramucirumab (full study population, *N* = 17 [post-ICI] vs *N* = 5 [ICI-naive]; propensity score-matched cohort, *N* = 12 [post-ICI] vs *N* = 5 [ICI-naive]) and bevacizumab (full study population, *N* = 0 [post-ICI] vs *N* = 9 [ICI-naive]; propensity score-matched cohort, *N* = 0 [post-ICI] vs *N* = 7 [ICI-naive])

COPD: chronic obstructive pulmonary disease; ECOG: Eastern Cooperative Oncology Group; ICI: immune checkpoint inhibitor; ILD: interstitial lung disease; irAEs: immune-related adverse events; NA: not applicable; PS: performance status; VEGF: vascular endothelial growth factor; VEGF-R: vascular endothelial growth factor-receptor

## Discussion

With ICI indications continuing to increase, attention has been paid to the durable effects of ICIs that predispose patients receiving prior ICI therapy to potentially increased risk and severity of irAEs during subsequent molecular targeted treatment [9, 12–17]. However, there is a dearth of knowledge as to whether the incidence and severity of DIILD are influenced by the prolonged nontumor-specific immune activation resulting from prior ICIs during subsequent systemic antineoplastic treatment in general or in a specific context according to the cancer type and therapeutic agent. In this study, we observed a higher cumulative incidence of DIILD in patients with lung cancer compared with those with other cancers. We also revealed that, among anticancer agents administered to lung cancer patients, docetaxel was the most common cause of DIILD. Of potential importance, docetaxel-induced ILD incidence was numerically higher in the post-ICI setting compared with that in ICI-naïve NSCLC patients, although there was no significant difference.

Our finding that lung cancer patients are at unique risk of DIILD is in keeping with previous literature, and has been suggested to result from multiple factors including a pulmonary tumor burden affecting the microenvironment and inflammatory response, exposure to tobacco smoke, and the underlying lung status [8, 24–26]. In the present study, several factors, such as chronic obstructive pulmonary disease, pleural fluid, and a history of prior thoracic radiotherapy, were more common in patients with lung cancer compared with other types of cancer, although patients with preexisting ILD appeared to be more strictly excluded from the use of ICIs in lung cancer patients.

In this study, docetaxel was the most common cause of DIILD in patients with NSCLC in the post-ICI setting. Since chemo-IO as first-line treatment for NSCLC [27–29] was designated a standard of care, docetaxel is frequently used as the second-line treatment after prior use of ICIs. Furthermore, ICIs have also been used as second-line treatment after platinum-based chemotherapy based on landmark trials [30–33], and docetaxel is an option after ICI failure in this setting. Therefore, patients with NSCLC are likely to receive docetaxel after the use of ICIs, which should be considered when interpreting our results. In the present study, there was no significant difference in cumulative incidence of ILD between NSCLC patients receiving docetaxel or other cytotoxic regimens (Additional file 2: Fig. S2a), which was partly because of the small number of patients. Nevertheless, the identification of docetaxel as an inducer of ILD is supported by previous reports showing that the rate of grade 3 or 4 ILD caused by docetaxel ranged from 7 to 47% in NSCLC patients, which appeared to depend on several factors including total dose and concomitant treatment with other agents and radiotherapy [34], and that docetaxel is one of the cytotoxic agents most likely to cause acute exacerbations of ILD with lung cancer [35, 36]. In the present study, all the docetaxel-induced ILDs in NSCLC patients developed within 4 months of treatment initiation (median [range], 52 days [9–120]), which suggests a need for close monitoring in the early phase of treatment.

One of the notable findings of this study was that prior ICIs might have been associated with increased risk of ILD when docetaxel was subsequently used in patients with lung cancer. This is supported by another Japanese study reporting that combination therapy with docetaxel and ramucirumab, an anti-VEGFR-2 monoclonal antibody, tended to increase pneumonitis in ICI-pretreated compared with ICI-naïve NSCLC patients (17% vs 4.8%) [37]. As Japanese patients are susceptible to DIILD [38], it might not be possible to generalize these observations among different ethnicities. Furthermore, it should be taken into consideration that the difference in the cumulative incidence of docetaxel-induced ILD between post-ICI and ICI-naïve settings in the present study was not significant. This could have partly been because of the limited patient numbers, and this requires further well-designed studies.

Based on the results in the REVEL study showing the improved survival of NSCLC patients receiving ramucirumab plus docetaxel who had progressed during or after first-line platinum-based chemotherapy as compared with placebo plus docetaxel [39], ramucirumab has been favorably used with docetaxel. In the phase III RELAY study, the incidence of ILD was reported to be lower in the ramucirumab plus erlotinib arm than in the placebo plus erlotinib arm [40]. Similarly, in the phase III NEJ026 trial, no patients developed ILD in the bevacizumab (an anti-VEGF monoclonal antibody) plus erlotinib arm, compared with 4% in the erlotinib monotherapy arm [41]. The incidence of ILD was also reported to be lower in the osimertinib plus bevacizumab arm (3%) than in the osimertinib monotherapy arm (18%) in the randomized phase II WJOG9717L study [42]. Furthermore, bevacizumab might potentially prevent the development of acute exacerbation of ILD in patients with nonsquamous NSCLC [43]. However, although the incidence of docetaxel-induced ILD was numerically higher in the post-ICI group than in the ICI-naïve group in the present study, NSCLC patients receiving docetaxel were more frequently treated with concomitant VEGF/VEGFR inhibitors in the post-ICI setting (60.7% vs 25.5%; Table 3). Given the potential of VEGF/VEGFR inhibitors to inhibit the development of DIILD, we adjusted this imbalance by propensity score matching. Additionally, in our study no patients with NSCLC received nintedanib, an intracellular inhibitor that targets multiple tyrosine kinases including VEGF, FGF, and PDGF receptors [44], in combination with docetaxel, because this combination therapy has yet to be approved in Japan. Whether antiangiogenic agents possess a protective ability against docetaxel-induced ILD, particularly in the post-ICI setting, warrants further study.

There are several limitations of this study. This was a retrospective study conducted at a single center. However, it would be difficult to prospectively compare the incidence of DIILD according to the presence of prior immunotherapy in the current IO era. The variable duration of prior ICI therapy in this study should also be acknowledged. Whether dose or duration of prior ICI therapy is associated with an increased risk of post-ICI DIILD requires larger, prospective studies. Another limitation is the relatively small numbers of individual cancer types. In addition, a small number of DIILD events (*N* = 4) in the ICI-naïve NSCLC cohort precludes solid comparisons between post-ICI and ICI-naïve patients, particularly after matching. Furthermore, the proportions of concomitant use of VEGF/VEGFR inhibitors with docetaxel in NSCLC patients were not balanced between the post-ICI and ICI-naïve groups, likely because of the proximity of the approval dates of ramucirumab and ICIs. Nevertheless, we attempted to eliminate bias in the patient selection using a propensity score matching method. Finally, the true causative relationships of identified agents with ILD were hardly to be proved, particularly considering the specific background in this study as the post-ICI setting where ICIs as well as other agents used in prior treatment lines should have played some roles for the development of ILD.

## Conclusions

Our results suggest that patients with lung cancer, compared with a range of other types of cancer, may uniquely have a high risk of developing DIILD in subsequent regimens after ICI treatment. Whether NSCLC patients are predisposed to an additional risk of docetaxel-induced ILD by prior ICIs should be addressed in further studies, because our findings are hypothesis-generating. Those studies, together with our results, would enable more accurate clinical risk–benefit assessment for DIILD in cancer patients who are due to receive subsequent systemic therapy after failure of ICIs.

### Supplementary Information

**Additional file 1: Figure S1.** Chest CT images showing preexisting interstitial lung disease (ILD) and drug-induced ILD (DIILD) at the post-ICI setting. Each case number corresponds to that in Table S2. In case 11 (a man with non-small cell lung cancer), CT before initiation of docetaxel therapy following prior durvalumab monotherapy showed localized subpleural reticulation in the lower right lobe (**a**). CT at the onset of DIILD demonstrated new diffuse ground-glass opacity (GGO) (**b**). In case 12 (a man with bladder cancer), CT before initiation of enfortumab vedotin therapy following prior pembrolizumab monotherapy showed bilateral peripheral linear shadows with slight GGO (**c**). CT at the time of DIILD diagnosis showed extensive bilateral areas of GGO and airspace consolidation with traction bronchiectasis (**d**). In case 14 (a man with esophageal cancer), CT before initiation of docetaxel therapy following prior nivolumab monotherapy showed slight subpleural reticulation and GGO with interlobular septal thickening in the right lower lobe (**e**). CT image at the onset of DIILD demonstrated multifocal patchy alveolar opacities (**f**).

**Additional file 2: Figure S2.** Cumulative incidence of the risk of developing interstitial lung disease caused by cytotoxic chemotherapy alone or molecular targeted therapy with or without chemotherapy in cancer patients after receiving immune checkpoint inhibitors.

**Additional file 3: Figure S3.** Cumulative incidence of the risk of developing cytotoxic chemotherapy-induced interstitial lung disease (ILD) in patients with non-small cell lung cancer after receiving immune checkpoint inhibitors.** a** Cumulative incidence of ILD according to treatment regimen (**a**, docetaxel vs other cytotoxic regimens; **b**, taxane-based vs non-taxane-based cytotoxic regimens).

**Additional file 4: Figure S4.** Cumulative incidence function for the risk of developing docetaxel-induced interstitial lung disease in patients with non-small cell lung cancer at the post-immune checkpoint inhibitor (ICI) or ICI-naïve setting.

**Additional file 5: Table S1.** Patient characteristics according to post-ICI drug-induced interstitial lung disease.

**Additional file 6: Table S2.** Clinical characteristics of patients with drug-induced interstitial lung disease caused by post-ICI antineoplastic therapy (*N* = 14).

**Additional file 7: Table S3.** Antineoplastic agents administered immediately following prior ICI-containing regimens.

**Additional file 8: Table S4.** Patient characteristics according to cancer type (lung cancer vs others).

## Data Availability

The datasets generated during and/or analyzed during the current study are available from the corresponding author on reasonable request.

## Abbreviations

CI: Confidence interval
CTLA-4: Cytotoxic T-lymphocyte-associated antigen 4
DAD: Diffuse alveolar damage
DIILD: Drug-induced ILD
ECOG: Eastern Cooperative Oncology Group
EGFR: Epidermal growth factor receptor
HP: Hypersensitivity pneumonia
HR: Hazard ratio
HRCT: High-resolution computed tomography
ICI: Immune checkpoint inhibitor
ILD: Interstitial lung disease
IO: Immuno-oncology
irAE: Immune-related adverse event
NSCLC: Non-small cell lung cancer
NSIP: Nonspecific interstitial pneumonia
OP: Organizing pneumonia
OS: Overall survival
PD-1: Programmed death-1
PD-L1: Programmed death-ligand 1
PEo: Simple pulmonary eosinophilia
PFS: Progression-free survival
PS: Performance status
SCLC: Small cell lung cancer
VEGF: Vascular endothelial growth factor
VEGFR: VEGF-receptor

## References

[1] Haslam A, Prasad V (2019). Estimation of the percentage of US patients with cancer who are eligible for and respond to checkpoint inhibitor immunotherapy drugs. JAMA Netw Open.

[2] Suresh K, Psoter KJ, Voong KR (2019). Impact of checkpoint inhibitor pneumonitis on survival in NSCLC patients receiving immune checkpoint immunotherapy. J Thorac Oncol.

[3] Suzuki Y, Karayama M, Uto T (2020). Assessment of immune-related interstitial lung disease in patients with NSCLC treated with immune checkpoint inhibitors: a multicenter prospective study. J Thorac Oncol.

[4] Inoue Y, Inui N, Karayama M (2023). Serum immune modulators associated with immune-related toxicities and efficacy of atezolizumab in patients with non-small cell lung cancer. J Cancer Res Clin Oncol.

[5] Brahmer JR, Lacchetti C, Schneider BJ (2018). Management of immune-related adverse events in patients treated with immune checkpoint inhibitor therapy: American Society of Clinical Oncology Clinical Practice Guideline. J Clin Oncol.

[6] Naidoo J, Wang X, Woo KM (2017). Pneumonitis in patients treated with anti-programmed death-1/programmed death ligand 1 therapy. J Clin Oncol.

[7] Ikeda S, Kato T, Kenmotsu H (2020). A phase 2 study of atezolizumab for pretreated NSCLC with idiopathic interstitial pneumonitis. J Thorac Oncol.

[8] Nishino M, Giobbie-Hurder A, Hatabu H, Ramaiya NH, Hodi FS (2016). Incidence of programmed cell death 1 inhibitor-related pneumonitis in patients with advanced cancer: a systematic review and meta-analysis. JAMA Oncol.

[9] Oshima Y, Tanimoto T, Yuji K, Tojo A (2018). EGFR-TKI-associated interstitial pneumonitis in nivolumab-treated patients with non-small cell lung cancer. JAMA Oncol.

[10] Oxnard GR, Yang JC, Yu H (2020). TATTON: a multi-arm, phase Ib trial of osimertinib combined with selumetinib, savolitinib, or durvalumab in EGFR-mutant lung cancer. Ann Oncol.

[11] Ahn MJ, Cho BC, Ou X (2022). Osimertinib plus durvalumab in patients with EGFR-mutated, advanced NSCLC: a phase 1b, open-label. Multicenter Trial J Thorac Oncol.

[12] Schoenfeld AJ, Arbour KC, Rizvi H (2019). Severe immune-related adverse events are common with sequential PD-(L)1 blockade and osimertinib. Ann Oncol.

[13] McCoach CE, Rolfo C, Drilon A (2022). Hypersensitivity reactions to selpercatinib treatment with or without prior immune checkpoint inhibitor therapy in patients with NSCLC in LIBRETTO-001. J Thorac Oncol.

[14] Lin JJ, Chin E, Yeap BY (2019). Increased hepatotoxicity associated with sequential immune checkpoint inhibitor and crizotinib therapy in patients with non-small cell lung cancer. J Thorac Oncol.

[15] Harding JJ, Pulitzer M, Chapman PB (2012). Vemurafenib sensitivity skin reaction after ipilimumab. N Engl J Med.

[16] Imafuku K, Yoshino K, Ymaguchi K, Tsuboi S, Ohara K, Hata H (2017). Nivolumab therapy before vemurafenib administration induces a severe skin rash. J Eur Acad Dermatol.

[17] Dimitriou F, Matter AV, Mangana J (2019). Cytokine release syndrome during sequential treatment with immune checkpoint inhibitors and kinase inhibitors for metastatic melanoma. J Immunother.

[18] Kaku S, Horinouchi H, Watanabe H (2022). Incidence and prognostic factors in severe drug-induced interstitial lung disease caused by antineoplastic drug therapy in the real world. J Cancer Res Clin Oncol.

[19] Wekking D, Porcu M, Pellegrino B (2023). Multidisciplinary clinical guidelines in proactive monitoring, early diagnosis, and effective management of trastuzumab deruxtecan (T-DXd)-induced interstitial lung disease (ILD) in breast cancer patients. ESMO Open.

[20] Yamaguchi O, Kaira K, Kawasaki T (2020). Severe hepatotoxicity due to osimertinib after nivolumab therapy in patients with non-small cell lung cancer harboring EGFR mutation. Thorac Cancer.

[21] Osa A, Uenami T, Koyama S (2018). Clinical implications of monitoring nivolumab immunokinetics in non-small cell lung cancer patients. JCI Insight.

[22] Johkoh T, Lee KS, Nishino M (2021). Chest CT diagnosis and clinical management of drug-related pneumonitis in patients receiving molecular targeting agents and immune checkpoint inhibitors a position paper from the Fleischner Society. Chest.

[23] Kanda Y (2013). Investigation of the freely available easy-to-use software 'EZR' for medical statistics. Bone Marrow Transplant.

[24] Moey MYY, Gougis P, Goldschmidt V (2020). Increased reporting of fatal pneumonitis associated with immune checkpoint inhibitors: a WHO pharmacovigilance database analysis. Eur Respir J.

[25] Delaunay M, Cadranel J, Lusque A (2017). Immune-checkpoint inhibitors associated with interstitial lung disease in cancer patients. Eur Respir J.

[26] Ma K, Lu Y, Jiang S, Tang J, Li X, Zhang Y (2018). The relative risk and incidence of immune checkpoint inhibitors related pneumonitis in patients with advanced cancer: a meta-analysis. Front Pharmacol.

[27] Gandhi L, Rodriguez-Abreu D, Gadgeel S (2018). Pembrolizumab plus chemotherapy in metastatic non-small-cell lung cancer. N Engl J Med.

[28] Paz-Ares L, Luft A, Vicente D (2018). Pembrolizumab plus chemotherapy for squamous non-small-cell lung cancer. N Engl J Med.

[29] Socinski MA, Jotte RM, Cappuzzo F (2018). Atezolizumab for first-line treatment of metastatic nonsquamous NSCLC. N Engl J Med.

[30] Brahmer J, Reckamp KL, Baas P (2015). Nivolumab versus docetaxel in advanced squamous-cell non-small-cell lung cancer. N Engl J Med.

[31] Borghaei H, Paz-Ares L, Horn L (2015). Nivolumab versus docetaxel in advanced nonsquamous non-small-cell lung cancer. N Engl J Med.

[32] Rittmeyer A, Barlesi F, Waterkamp D (2017). Atezolizumab versus docetaxel in patients with previously treated non-small-cell lung cancer (OAK): a phase 3, open-label, multicentre randomised controlled trial. Lancet.

[33] Herbst RS, Baas P, Kim DW (2016). Pembrolizumab versus docetaxel for previously treated, PD-L1-positive, advanced non-small-cell lung cancer (KEYNOTE-010): a randomised controlled trial. Lancet.

[34] Grande C, Villanueva MJ, Huidobro G, Casal J (2007). Docetaxel-induced interstitial pneumonitis following non-small-cell lung cancer treatment. Clin Transl Oncol.

[35] Kenmotsu H, Naito T, Kimura M (2011). The risk of cytotoxic chemotherapy-related exacerbation of interstitial lung disease with lung cancer. J Thorac Oncol.

[36] Kanaji N, Tadokoro A, Kita N (2016). Impact of idiopathic pulmonary fibrosis on advanced non-small cell lung cancer survival. J Cancer Res Clin Oncol.

[37] Harada D, Takata K, Mori S (2019). Previous immune checkpoint inhibitor treatment to increase the efficacy of docetaxel and ramucirumab combination chemotherapy. Anticancer Res.

[38] Kudoh S, Kato H, Nishiwaki Y (2008). Interstitial lung disease in Japanese patients with lung cancer: a cohort and nested case-control study. Am J Respir Crit Care Med.

[39] Garon EB, Ciuleanu TE, Arrieta O (2014). Ramucirumab plus docetaxel versus placebo plus docetaxel for second-line treatment of stage IV non-small-cell lung cancer after disease progression on platinum-based therapy (REVEL): a multicentre, double-blind, randomised phase 3 trial. Lancet.

[40] Nishio M, Seto T, Reck M (2020). Ramucirumab or placebo plus erlotinib in EGFR-mutated, metastatic non-small-cell lung cancer: East Asian subset of RELAY. Cancer Sci.

[41] Saito H, Fukuhara T, Furuya N (2019). Erlotinib plus bevacizumab versus erlotinib alone in patients with EGFR-positive advanced non-squamous non-small-cell lung cancer (NEJ026): interim analysis of an open-label, randomised, multicentre, phase 3 trial. Lancet Oncol.

[42] Kenmotsu H, Wakuda K, Mori K (2022). Randomized phase 2 study of osimertinib plus bevacizumab versus osimertinib for untreated patients with nonsquamous NSCLC harboring EGFR mutations: WJOG9717L Study. J Thorac Oncol.

[43] Hamada S, Ichiyasu H, Ikeda T (2019). Protective effect of bevacizumab on chemotherapy-related acute exacerbation of interstitial lung disease in patients with advanced non-squamous non-small cell lung cancer. BMC Pulm Med.

[44] Hilberg F, Roth GJ, Krssak M (2008). BIBF 1120: triple angiokinase inhibitor with sustained receptor blockade and good antitumor efficacy. Cancer Res.

[45] von Elm E, Altman DG, Egger M (2007). The Strengthening the Reporting of Observational Studies in Epidemiology (STROBE) statement: guidelines for reporting observational studies. PLoS Med.

