# Supplementary figures and images for: Pan-cancer assessment of antineoplastic therapy-induced interstitial lung disease in patients receiving subsequent therapy immediately following immune checkpoint blockade therapy
Source: Respir Res. 2024 Jan 10;25:25. doi: 10.1186/s12931-024-02683-8 (PMC10777633; doi:10.1186/s12931-024-02683-8)

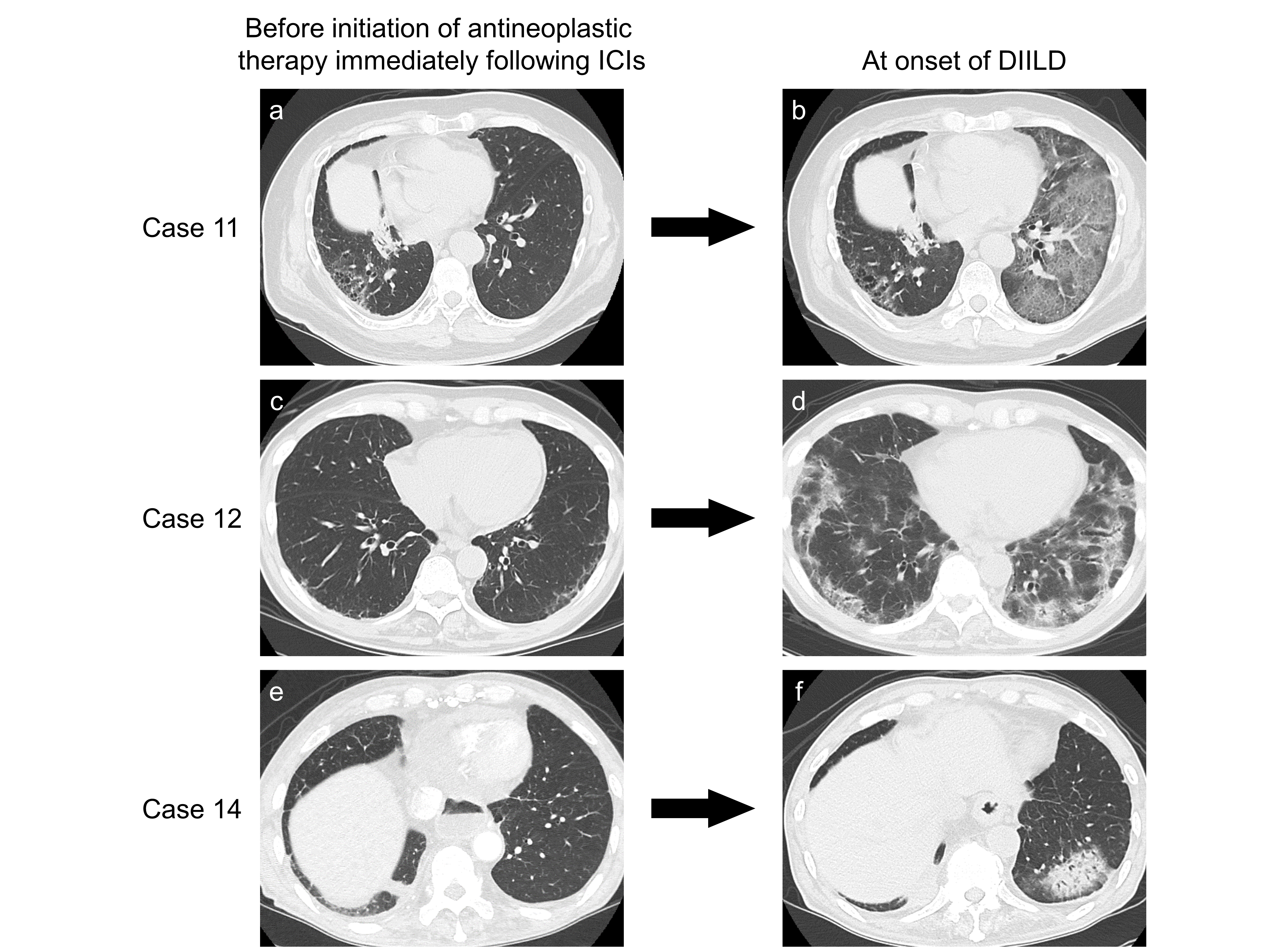

Supplement: Supplementary file 1 — Additional file 1: Figure S1. Chest CT images showing preexisting interstitial lung disease (ILD) and drug-induced ILD (DIILD) at the post-ICI setting. Each case number corresponds to that in Table S2. In case 11 (a man with non-small cell lung cancer), CT before initiation of docetaxel therapy following prior durvalumab monotherapy showed localized subpleural reticulation in the lower right lobe (a). CT at the onset of DIILD demonstrated new diffuse ground-glass opacity (GGO) (b). In case 12 (a man with bladder cancer), CT before initiation of enfortumab vedotin therapy following prior pembrolizumab monotherapy showed bilateral peripheral linear shadows with slight GGO (c). CT at the time of DIILD diagnosis showed extensive bilateral areas of GGO and airspace consolidation with traction bronchiectasis (d). In case 14 (a man with esophageal cancer), CT before initiation of docetaxel therapy following prior nivolumab monotherapy showed slight subpleural reticulation and GGO with interlobular septal thickening in the right lower lobe (e). CT image at the onset of DIILD demonstrated multifocal patchy alveolar opacities (f). [file 12931_2024_2683_MOESM1_ESM.tif]

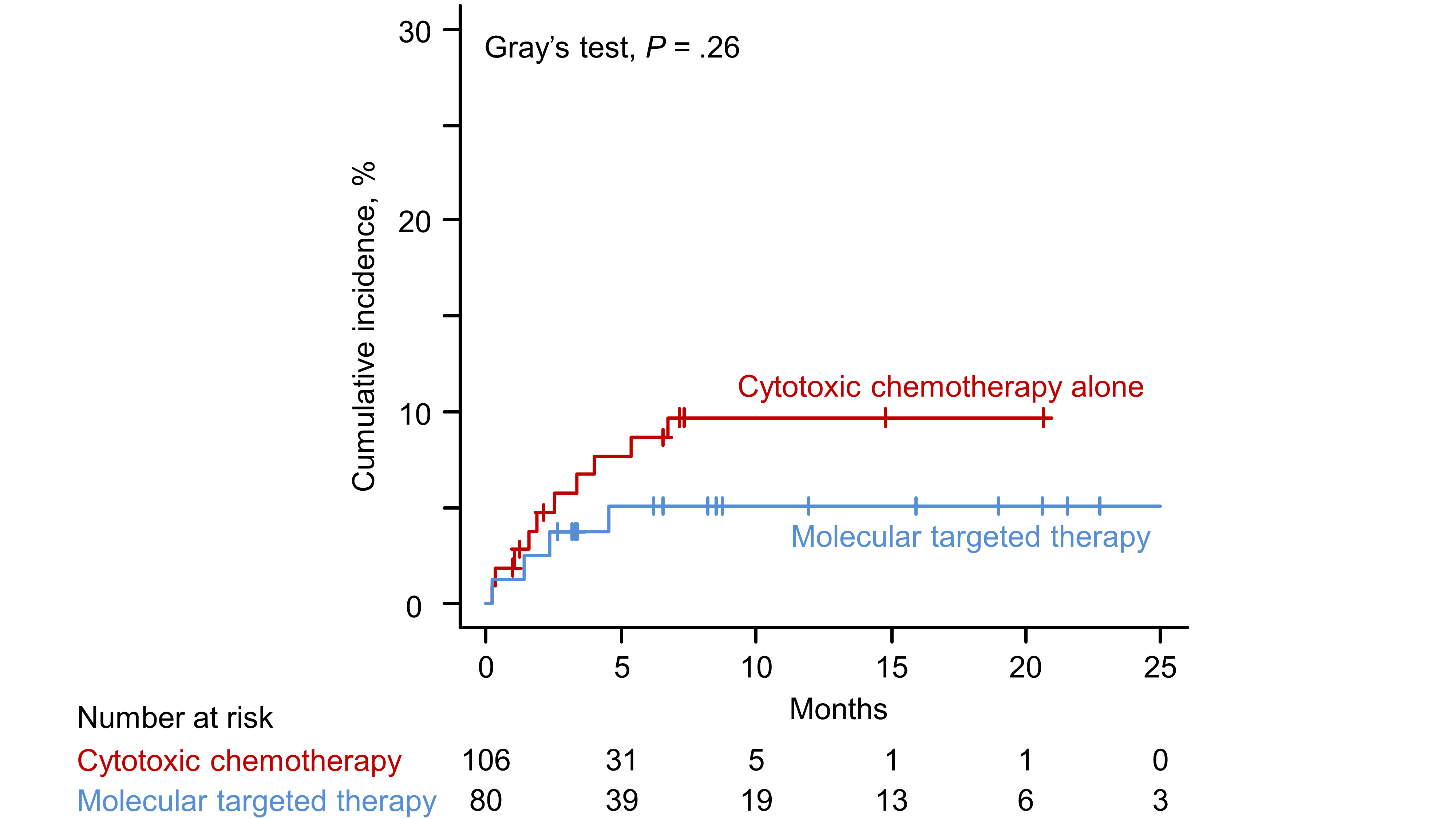

Supplement: Supplementary file 2 — Additional file 2: Figure S2. Cumulative incidence of the risk of developing interstitial lung disease caused by cytotoxic chemotherapy alone or molecular targeted therapy with or without chemotherapy in cancer patients after receiving immune checkpoint inhibitors. [file 12931_2024_2683_MOESM2_ESM.tif]

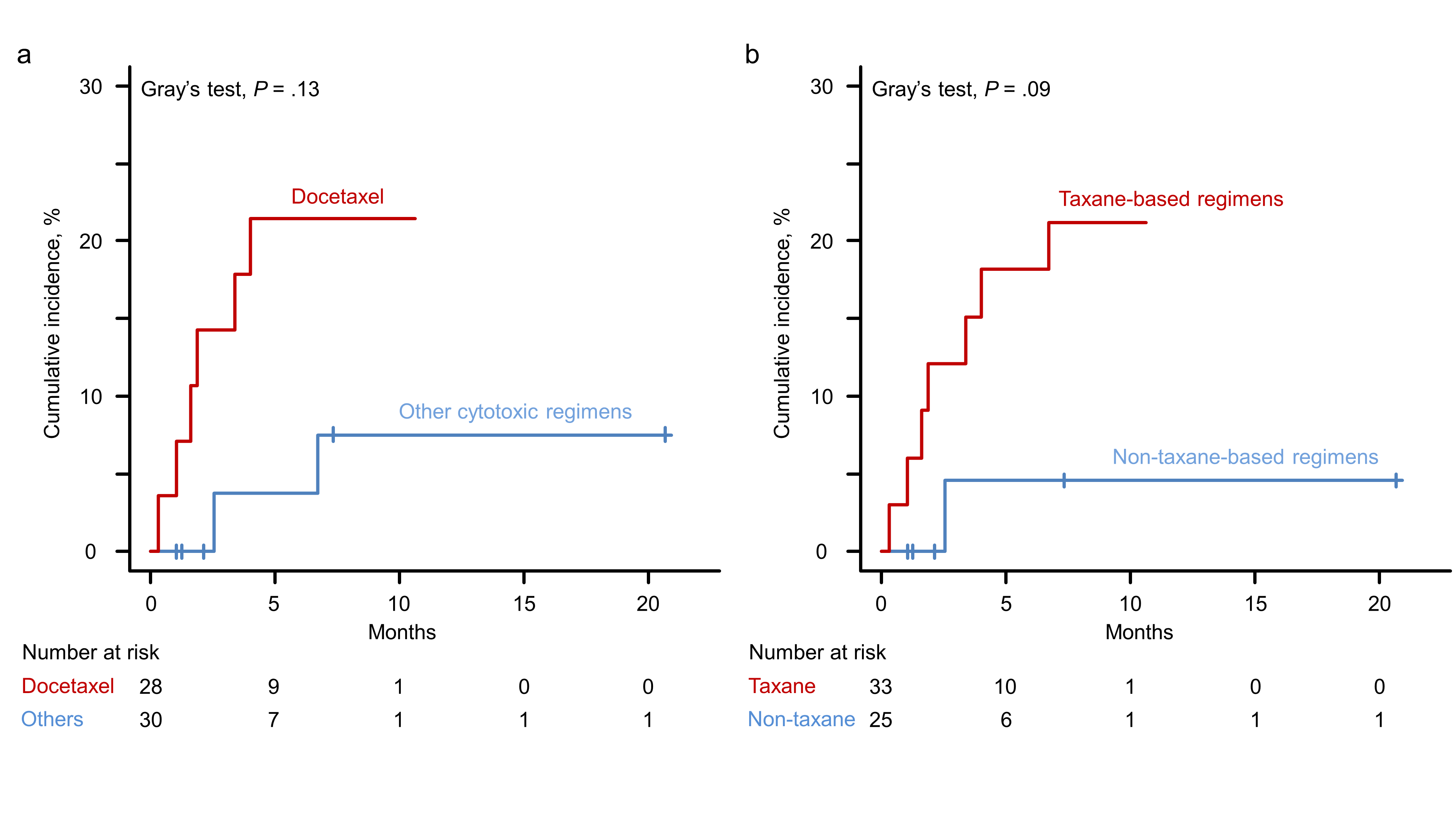

Supplement: Supplementary file 3 — Additional file 3: Figure S3. Cumulative incidence of the risk of developing cytotoxic chemotherapy-induced interstitial lung disease (ILD) in patients with non-small cell lung cancer after receiving immune checkpoint inhibitors. a Cumulative incidence of ILD according to treatment regimen (a, docetaxel vs other cytotoxic regimens; b, taxane-based vs non-taxane-based cytotoxic regimens). [file 12931_2024_2683_MOESM3_ESM.tif]

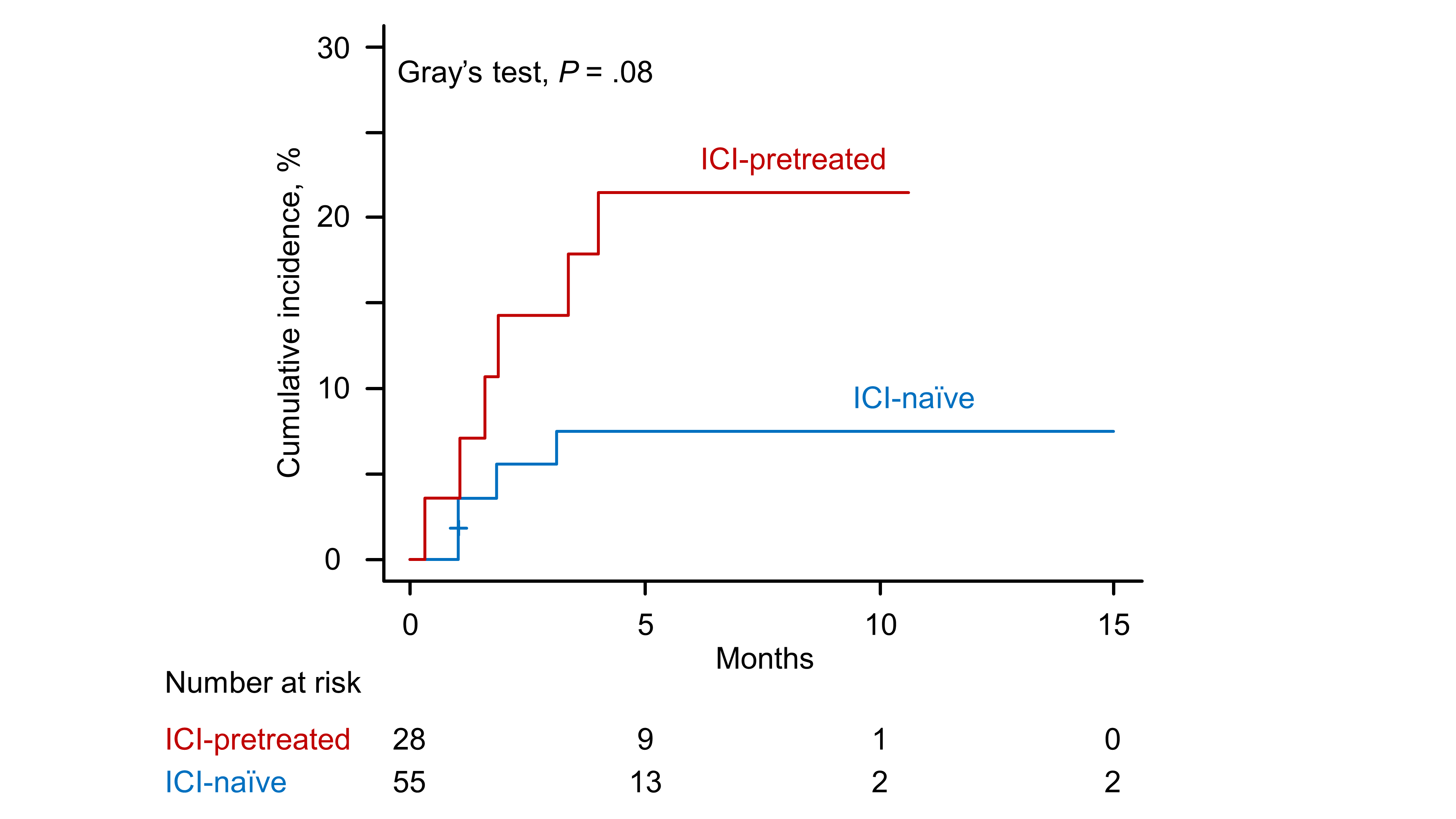

Supplement: Supplementary file 4 — Additional file 4: Figure S4. Cumulative incidence function for the risk of developing docetaxel-induced interstitial lung disease in patients with non-small cell lung cancer at the post-immune checkpoint inhibitor (ICI) or ICI-naïve setting. [file 12931_2024_2683_MOESM4_ESM.tif]
