# Supplementary material for: Pan-cancer assessment of antineoplastic therapy-induced interstitial lung disease in patients receiving subsequent therapy immediately following immune checkpoint blockade therapy
Source: Respir Res. 2024 Jan 10;25:25. doi: 10.1186/s12931-024-02683-8 (PMC10777633; doi:10.1186/s12931-024-02683-8)
Supplement: Supplementary file 5 — Additional file 5: Table S1. Patient characteristics according to post-ICI drug-induced interstitial lung disease. [file 12931_2024_2683_MOESM5_ESM.docx]

|  | Patients, No. (%) | |  |
| --- | --- | --- | --- |
|  | DIILD  (*N* = 14) | Non-DIILD  (*N* = 172) | *P*-value |
| Age, median (range), y | 67 (55–78) | 69 (32–83) | .33 |
| Sex |  |  |  |
| Male | 12 (85.7) | 131 (76.2) | .52 |
| Female | 2 (14.3) | 41 (23.8) |  |
| ECOG-PS |  |  |  |
| 0–1 | 10 (71.4) | 124 (72.1) | >.99 |
| ≥2 | 4 (28.6) | 48 (27.9) |  |
| Smoking status |  |  |  |
| Never | 4 (28.6) | 32 (18.6) | .33 |
| Current or former | 10 (71.4) | 120 (69.8) |  |
| Unknown | 0 (0) | 20 (11.6) |  |
| Comorbidity |  |  |  |
| Hypertension | 7 (50.0) | 59 (34.3) | .25 |
| Diabetes mellitus | 2 (14.3) | 37 (21.5) | .73 |
| COPD | 0 (0) | 20 (11.6) | .37 |
| Pulmonary emphysema | 8 (57.1) | 69 (40.1) | .26 |
| Pleural fluid | 10 (71.4) | 62 (36.0) | .02 |
| ILD | 3 (21.4) | 22 (12.8) | .41 |
| Radiation pneumonitis | 2 (14.3) | 15 (8.7) | .62 |
| Cardiovascular disease | 1 (7.1) | 25 (14.5) | .69 |
| Renal disorder | 1 (7.1) | 7 (4.1) | .47 |
| Stage |  |  |  |
| Ⅲ | 2 (14.3) | 27 (15.7) | .92 |
| Ⅳ | 10 (71.4) | 106 (61.6) |  |
| Recurrence | 2 (14.3) | 39 (22.7) |  |
| Primary organ |  |  |  |
| Lung/pleura | 11 (78.6) | 63 (36.6) | .08 |
| Kidney/urinary tract | 1 (7.1) | 57 (33.1) |  |
| Skin | 0 (0) | 10 (5.8) |  |
| Gastrointestinal tract | 1 (7.1) | 27 (15.7) |  |
| Head and neck | 1 (7.1) | 15 (8.7) |  |
| History of prior thoracic radiotherapy | 3 (21.4) | 31 (18.1) | .72 |
| Prior PD-1/PD-L1 inhibitors |  |  |  |
| Nivolumab | 3 (21.4) | 86 (50.0) | .14 |
| Pembrolizumab | 8 (57.1) | 56 (32.6) |  |
| Atezolizumab | 2 (14.3) | 20 (11.6) |  |
| Durvalumab | 1 (7.1) | 5 (2.9) |  |
| Avelumab | 0 (0) | 5 (2.9) |  |
| Prior ICI treatment |  |  |  |
| Monotherapy | 7 (50.0) | 119 (69.2) | .07 |
| Immunochemotherapy | 7 (50.0) | 32 (18.6) |  |
| Combination with CTLA-4 blockade therapy with or without chemotherapy | 0 (0) | 7 (4.1) |  |
| Combination with molecular targeted therapy | 0 (0) | 14 (8.1) |  |
| Duration of ICI therapy, median (range), m | 2.1 (0.5–7.5) | 3.1 (0.5–39.9) | .08 |
| irAEs in prior ICI regimens |  |  |  |
| ICI-induced ILD | 3 (21.4) | 15 (8.7) | .14 |
| Others | 4 (28.6) | 58 (33.7) | .78 |
| Post-ICI antineoplastic therapy |  |  |  |
| Cytotoxic chemotherapy | 11 (78.6) | 100 (58.1) | .16 |
| Molecular targeted therapy | 3 (21.4) | 72 (41.9) |  |
| Treatment line |  |  |  |
| 2nd | 9 (64.3) | 60 (34.9) | .04 |
| 3rd | 1 (7.1) | 60 (34.9) |  |
| ≥4th | 4 (28.6) | 52 (30.2) |  |
| Duration of post-ICI antineoplastic therapy, median (range), m | 2.1 (0.5–7.5) | 3.1 (0.5–39.9) | .08 |

COPD, chronic obstructive pulmonary disease; CTLA-4, cytotoxic T-lymphocyte-associated protein 4; DIILD, drug-induced interstitial lung disease; ECOG, Eastern Cooperative Oncology Group; ICI, immune checkpoint inhibitor; ILD, interstitial lung disease; irAEs, immune-related adverse events; PD-1, programmed death-1; PD-L1, programmed death-ligand 1; PS, performance status.
