# Supplementary material for: Pan-cancer assessment of antineoplastic therapy-induced interstitial lung disease in patients receiving subsequent therapy immediately following immune checkpoint blockade therapy
Source: Respir Res. 2024 Jan 10;25:25. doi: 10.1186/s12931-024-02683-8 (PMC10777633; doi:10.1186/s12931-024-02683-8)
Supplement: Supplementary file 6 — Additional file 6: Table S2. Clinical characteristics of patients with drug-induced interstitial lung disease caused by post-ICI antineoplastic therapy (N = 14). [file 12931_2024_2683_MOESM6_ESM.docx]

**Table S2. Clinical characteristics of patients with drug-induced interstitial lung disease caused by post-ICI antineoplastic therapy (*N* = 14)**

|  |  |  |  |  |  |  |  |  |  | Post-ICI treatment setting | | | | | | | |
| --- | --- | --- | --- | --- | --- | --- | --- | --- | --- | --- | --- | --- | --- | --- | --- | --- | --- |
| No. | Sex | Smoking | Primary organ | Stage | History of ILD | History of thoracic radiotherapy | Prior ICI | Prior ICI-ILD | Prior ICI-ILD,  grade^a^ | Age | Intervals from last ICI dose to post-ICI therapy, months | Regimen | ECOG-PS | Treatment line | Time to onset DIILD, days | DIILD pattern | DIILD grade^a^ |
| 1 | M | Yes | Lung | Ⅳ |  |  | Pemb | + | 2 | 68 | 5.5 | DTX/RAM | 2 | 2 | 9 | OP | 5 |
| 2 | M | Yes | Lung | Ⅳ |  |  | Pemb |  |  | 72 | 1.0 | DTX/RAM | 1 | 2 | 48 | PEo | 2 |
| 3 | M | No | Lung | R |  |  | Nivo |  |  | 67 | 1.0 | OSM | 2 | 10 | 7 | DAD | 5 |
| 4 | F | No | Lung | Ⅳ |  |  | Atez |  |  | 66 | 1.4 | DTX/RAM | 1 | 4 | 56 | HP | 1 |
| 5 | M | Yes | Lung | Ⅳ |  | + | Pemb | + | 3 | 69 | 2.3 | CBDCA/nabPTX | 1 | 2 | 201 | DAD | 3 |
| 6 | F | No | Lung | Ⅳ |  |  | Pemb |  |  | 78 | 5.5 | CDDP/PEM | 1 | 2 | 76 | OP | 3 |
| 7 | M | Yes | Lung | Ⅳ |  | + | Pemb | + | 2 | 60 | 2.2 | DTX/RAM | 1 | 2 | 120 | HP | 1 |
| 8 | M | Yes | Lung | Ⅳ |  |  | Pemb |  |  | 65 | 5.9 | DTX/RAM | 1 | 2 | 101 | HP | 2 |
| 9 | M | Yes | Lung | R |  |  | Nivo |  |  | 67 | 2.6 | OSM | 2 | 8 | 136 | DAD | 5 |
| 10 | M | Yes | Lung | Ⅳ |  |  | Atez |  |  | 62 | 1.8 | NGT | 2 | 2 | 6 | OP | 3 |
| 11 | M | Yes | Lung | Ⅲ | + | + | Durv |  |  | 59 | 0.9 | DTX | 1 | 2 | 31 | HP | 3 |
| 12 | M | Yes | Bladder | Ⅳ | + |  | Pemb |  |  | 69 | 0.5 | EV | 0 | 4 | 70 | DAD | 3 |
| 13 | M | No | Buccal mucosa | Ⅳ |  |  | Pemb |  |  | 55 | 0.7 | PTX/C-mab | 1 | 2 | 42 | OP | 1 |
| 14 | M | Yes | Esophagus | Ⅲ | + |  | Nivo |  |  | 66 | 0.5 | DTX | 0 | 3 | 161 | OP | 1 |

^a^The severity of ICI-ILD and DIILD was scored according to the Common Terminology Criteria for Adverse Events (CTCAE) version 5.0.

Atez, atezolizumab; CBDCA, carboplatin; CDDP, cisplatin; C-mab, cetuximab; DAD, diffuse alveolar damage; DIILD, drug-induced interstitial lung disease; DTX, docetaxel; Durv, durvalumab; ECOG, Eastern Cooperative Oncology Group; EV, enfortumab vedotin; F, female; HP, hypersensitivity pneumonia; ILD, interstitial lung disease; irAE, immune-related adverse events; M, male; NGT, nogitecan; Nivo, nivolumab; OP, organizing pneumonia; OSM, osimertinib; PEM, pemetrexed; Pemb, pembrolizumab; PEo, pulmonary eosinophilia; PS, performance status; PTX, paclitaxel; R, recurrence; RAM, ramucirumab.
