# Supplementary material for: Pan-cancer assessment of antineoplastic therapy-induced interstitial lung disease in patients receiving subsequent therapy immediately following immune checkpoint blockade therapy
Source: Respir Res. 2024 Jan 10;25:25. doi: 10.1186/s12931-024-02683-8 (PMC10777633; doi:10.1186/s12931-024-02683-8)
Supplement: Supplementary file 7 — Additional file 7: Table S3. Antineoplastic agents administered immediately following prior ICI-containing regimens. [file 12931_2024_2683_MOESM7_ESM.docx]

**Table S3. Antineoplastic agents administered immediately following prior ICI-containing regimens**

| Primary organ | Cytotoxic agents alone | No. of patients  (*N* = 106) | Molecular targeted therapy | No. of patients  (*N* = 80) |
| --- | --- | --- | --- | --- |
| Lung/pleura | Docetaxel (± platinum ± ramucirumab) | 28 | Afatinib | 3 |
|  | S-1 (± platinum) | 13 | Osimertinib | 3 |
|  | Pemetrexed (± platinum ± bevacizumab) | 8 | Crizotinib | 1 |
|  | Nab-paclitaxel (± platinum) | 4 | Erlotinib | 1 |
|  | Amrubicin | 3 | Gefitinib | 1 |
|  | Nogitecan | 2 | Necitumumab + CDDP + gemcitabine | 1 |
|  | Paclitaxel (± platinum) | 2 | Pimitespib | 1 |
|  | Vinorelbine | 2 |  |  |
|  | FOLFOX | 1 |  |  |
| Kidney/urinary tract | Gemcitabine + paclitaxel | 6 | Cabozantinib | 19 |
|  | Paclitaxel (± platinum) | 3 | Pazopanib | 9 |
|  | Gemcitabine (± platinum) | 2 | Enfortumab vedotin | 6 |
|  | Ifosfamide + doxorubicin | 1 | Axitinib | 5 |
|  | S-1 | 1 | Sunitinib | 4 |
|  |  |  | Sorafenib | 1 |
|  |  |  | Temsirolimus | 1 |
| Skin | CDDP + etoposide | 2 | Dabrafenib + trametinib | 5 |
|  |  |  | Encorafenib + binimetinib | 2 |
|  |  |  | Trastuzumab deruxtecan | 1 |
| Gastrointestinal tract | Docetaxel (± platinum + 5-fluorouracil) | 7 | Trastuzumab deruxtecan | 1 |
|  | FOLFOX | 6 |  |  |
|  | Trifluridine | 5 |  |  |
|  | Irinotecan | 4 |  |  |
|  | Paclitaxel | 2 |  |  |
|  | S-1 | 2 |  |  |
|  | Ramucirumab | 1 |  |  |
| Head and neck | S-1 | 1 | Paclitaxel + cetuximab | 15 |

CDDP, cisplatin; FOLFOX, folinic acid + 5-fluorouracil + oxaliplatin; ICI, immune checkpoint inhibitor.
