# Supplementary material for: Pan-cancer assessment of antineoplastic therapy-induced interstitial lung disease in patients receiving subsequent therapy immediately following immune checkpoint blockade therapy
Source: Respir Res. 2024 Jan 10;25:25. doi: 10.1186/s12931-024-02683-8 (PMC10777633; doi:10.1186/s12931-024-02683-8)
Supplement: Supplementary file 8 — Additional file 8: Table S4. Patient characteristics according to cancer type (lung cancer vs others). [file 12931_2024_2683_MOESM8_ESM.docx]

|  | Patients, No. (%) | |  |
| --- | --- | --- | --- |
|  | Lung cancer  (*N* = 73) | Others  (*N* = 113) | *P*-value |
| Age, median (range), y | 67 (40–83) | 69 (32–82) | .69 |
| Sex |  |  | .59 |
| Male | 58 (79.5) | 85 (75.2) |  |
| Female | 15 (20.5) | 28 (24.8) |  |
| ECOG-PS |  |  |  |
| 0 or 1 | 48 (65.8) | 86 (76.1) | .33 |
| ≥2 | 25 (34.2) | 27 (23.9) |  |
| Smoking status |  |  |  |
| Never | 13 (17.8) | 23 (20.4) | .34 |
| Current or former | 60 (82.2) | 70 (61.9) |  |
| Unknown | 0 (0) | 20 (17.7) |  |
| Comorbidity |  |  |  |
| Hypertension | 33 (45.2) | 33 (29.2) | .03 |
| Diabetes mellitus | 16 (21.9) | 23 (20.4) | .85 |
| COPD | 14 (19.2) | 6 (5.3) | .006 |
| Pulmonary emphysema | 35 (47.9) | 42 (37.2) | .17 |
| Pleural fluid | 41 (56.2) | 31 (27.4) | <.001 |
| ILD | 5 (6.8) | 20 (17.7) | .046 |
| Radiation pneumonitis | 10 (13.7) | 7 (6.2) | .11 |
| Cardiovascular disease | 13 (17.8) | 13 (11.5) | .28 |
| Renal disorder | 3 (4.1) | 5 (4.4) | >.99 |
| Stage |  |  |  |
| Ⅲ | 9 (12.3) | 20 (17.7) | .50 |
| Ⅳ | 49 (67.1) | 67 (59.3) |  |
| Recurrence | 15 (20.5) | 26 (23.0) |  |
| History of prior thoracic radiotherapy | 21 (28.8) | 13 (11.5) | .004 |
| Prior PD-1/PD-L1 inhibitors |  |  |  |
| Nivolumab | 20 (27.4) | 69 (61.1) | <.001 |
| Pembrolizumab | 25 (34.2) | 39 (34.5) |  |
| Atezolizumab | 22 (30.1) | 0 (0) |  |
| Durvalumab | 6 (8.2) | 0 (0) |  |
| Avelumab | 0 (0) | 5 (4.4) |  |
| Prior ICI treatment |  |  |  |
| Monotherapy | 39 (53.4) | 88 (77.9) | <.001 |
| Immunochemotherapy | 32 (43.8) | 7 (6.2) |  |
| Combination with CTLA-4 blockade therapy with or without chemotherapy | 2 (2.7) | 5 (4.4) |  |
| Combination with molecular targeted therapy | 0 (0) | 13 (11.5) |  |
| Duration of ICI therapy, median (range), m | 3.8 (0.5–52.5) | 4.1 (0.5–32.7) | .90 |
| irAEs in prior ICI regimens |  |  |  |
| ICI-induced ILD | 11 (15.1) | 7 (6.2) | .07 |
| Others | 22 (30.1) | 40 (35.4) | .53 |
| Post-ICI antineoplastic therapy |  |  |  |
| Cytotoxic chemotherapy | 64 (87.7) | 39 (34.5) | <.001 |
| Molecular targeted therapy | 9 (12.3) | 74 (65.5) |  |
| Treatment line |  |  |  |
| 2nd | 39 (53.4) | 30 (26.5) | <.001 |
| 3rd | 15 (20.5) | 46 (40.7) |  |
| ≥4th | 19 (26.0) | 37 (32.7) |  |
| Duration of post-ICI antineoplastic therapy, median (range), m | 2.7 (0.5–20.9) | 3.7 (0.5–39.9) | .003 |

COPD, chronic obstructive pulmonary disease; CTLA-4, cytotoxic T-lymphocyte-associated protein 4; DIILD, drug-induced interstitial lung disease; ECOG, Eastern Cooperative Oncology Group; ICI, immune checkpoint inhibitor; ILD, interstitial lung disease; irAEs, immune-related adverse events; PD-1, programmed death-1; PD-L1, programmed death-ligand 1; PS, performance status.
